# Supplementary material for: The Construction and Exploration of a Comprehensive MicroRNA Centered Regulatory Network in Foxtail Millet (Setaria italica L.)
Source: Front Plant Sci. 2022 May 6;13:848474. doi: 10.3389/fpls.2022.848474 (PMC9121102; doi:10.3389/fpls.2022.848474)
Supplement: Supplementary file 1 [file Data_Sheet_1.zip › Sit-miRN3023b_Seita.7G278200.1_908_TPlot.pdf]

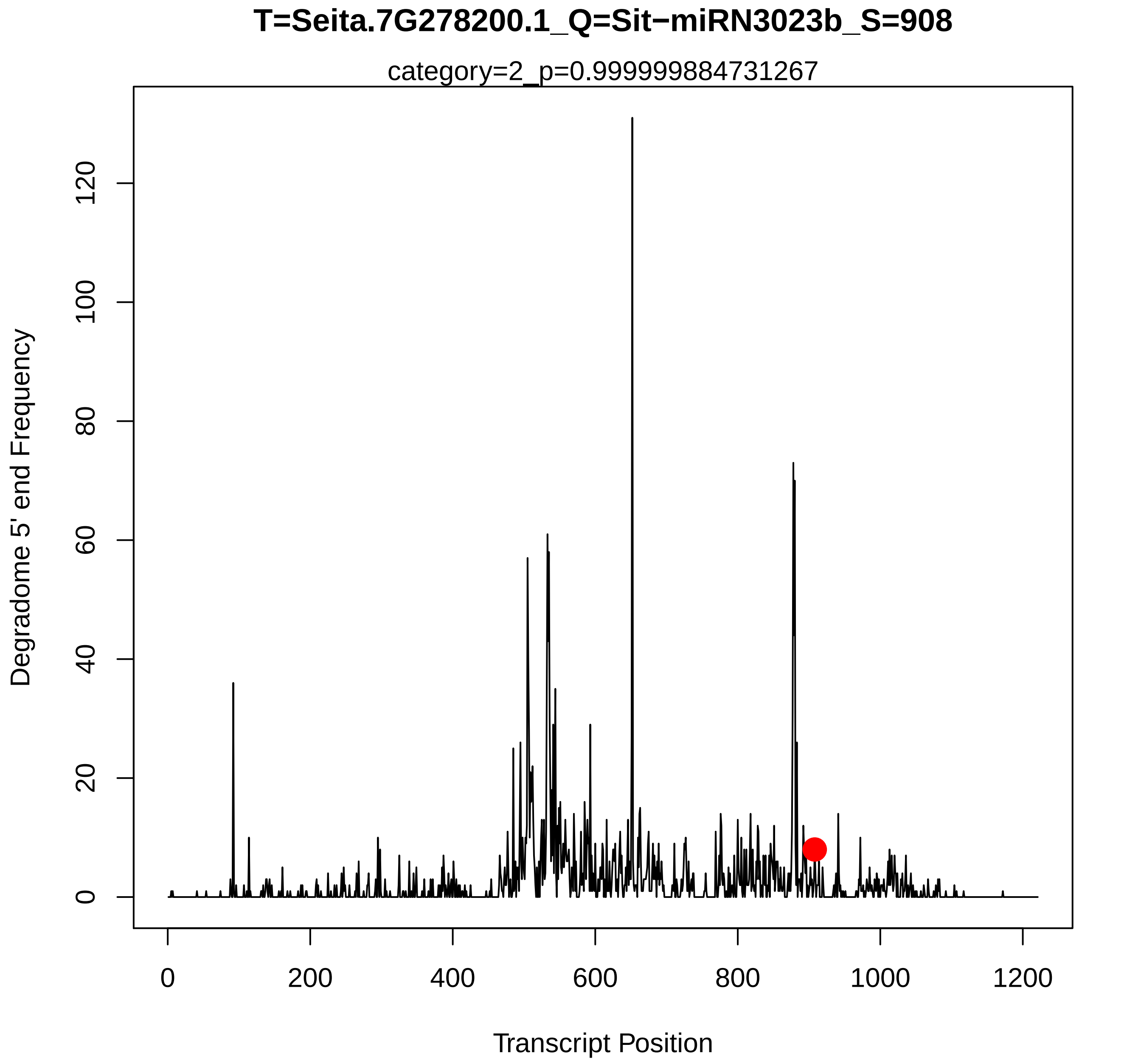
T=Seita.7G278200.1_Q=Sit−miRN3023b_S=908
category=2_p=0.999999884731267
Degradome 5' end Frequency
0
20
40
60
80
100
120
0
200
400
600
800
1000
1200
Transcript Position
